# Spatial asymmetries (“pseudoneglect”) in free visual exploration—modulation of age and relationship to line bisection

**DOI:** 10.1007/s00221-021-06165-x

**Published:** 2021-07-04

**Authors:** Kathrin Chiffi, Lorenzo Diana, Matthias Hartmann, Dario Cazzoli, Claudio L. Bassetti, René M. Müri, Aleksandra K. Eberhard-Moscicka

**Affiliations:** 1 Perception and Eye Movement Laboratory, Departments of Neurology and BioMedical Research, Inselspital, Bern University Hospital and University of Bern, Freiburgstarsse 46, 3010 Bern, Switzerland; 2 Department of Neurology, Inselspital, Bern University Hospital, Bern, Switzerland; 3 PhD Program in Neuroscience, School of Medicine and Surgery, University of Milano-Bicocca, Monza, Italy; 4 Faculty of Psychology, UniDistance Suisse, Brig, Switzerland; 5 Gerontechnology and Rehabilitation Group, University of Bern, Bern, Switzerland

**Keywords:** Pseudoneglect, Visual exploration, Eye movements, Line bisection

## Abstract

When humans visually explore an image, they typically tend to start exploring its left side. This phenomenon, so-called pseudoneglect, is well known, but its time-course has only sparsely been studied. Furthermore, it is unclear whether age influences pseudoneglect, and the relationship between visuo-spatial attentional asymmetries in a free visual exploration task and a classical line bisection task has not been established. To address these questions, 60 healthy participants, aged between 22 and 86, were assessed by means of a free visual exploration task with a series of naturalistic, colour photographs of everyday scenes, while their gaze was recorded by means of a contact-free eye-tracking system. Furthermore, a classical line bisection task was administered, and information concerning handedness and subjective alertness during the experiment was obtained. The results revealed a time-sensitive window during visual exploration, between 260 and 960 ms, in which age was a significant predictor of the leftward bias in gaze position, i.e., of pseudoneglect. Moreover, pseudoneglect as assessed by the line bisection task correlated with the average gaze position throughout a time-window of 300–1490 ms during the visual exploration task. These results suggest that age influences visual exploration and pseudoneglect in a time-sensitive fashion, and that the degree of pseudoneglect in the line bisection task correlates with the average gaze position during visual exploration in a time-sensitive manner.

## Introduction

Human visual exploration results from a complex interplay between saccadic eye movements and visual fixations. During the exploration of an image, saccades and fixations are typically not homogeneously distributed in space, but are driven by attention, saliency, and other cognitive factors. Healthy subjects may show a leftward bias in the initial phase of the visual exploration of an image (Dickinson and Intraub 2009; Foulsham 2013; Nuthmann and Matthias 2014; Ossandon et al. 2014; Hartmann, 2019). This so-called pseudoneglect has been interpreted as a small, but reliable, asymmetry in the distribution of attention, in which attention is preferentially directed towards the left side (Bowers and Heilman 1980; Jewell and McCourt 2000; Nicholls 2012; Thomas 2015). Pseudoneglect has been mainly reported in line bisection tasks and in visual exploration (Jewell and McCourt 2000; Foulsham et al. 2013; Nuthmann and Matthias 2014). Age may influence performance in such tasks, but the effect of age on pseudoneglect is still debated. While some studies reported a reduction or even a directional reversal of pseudoneglect in older healthy adults (Fujii 1995; Failla et al. 2003; Barrett and Craver-Lemley 2008; Schmitz and Peigneux 2011; Benwell^ 2014^), other studies reported no effect of age on this leftward bias; or even a stronger leftward bias with increasing age (De Agostini^ 1999^; Varnava and Halligan 2007; Brooks^ 2016^; Friedrich et al. 2016). Furthermore, other factors such as alertness (Paladini^ 2017^), gender (Friedrich et al. 2018), or handedness (Ossandon et al. 2014) may influence pseudoneglect.

Previous literature indicated that the pseudoneglect arises due to a hemispherical asymmetry between the left hemisphere and the right hemisphere, with visuospatial attention being controlled by frontal–parietal networks. The asymmetry is biased towards the left visual field due to a higher activity of the right hemisphere (Heilman and Van Den Abell 1980; Mesulam 1999; Corbetta and Shulman 2002). Using imaging techniques, such as MRI, it has, furthermore, been shown that for cognitive domains the lateralization of the brain activity is reduced with age and in general more bilateral (Dolcos et al. 2002; Brooks et al. 2016; Ng^ 2016^). This can thus also lead to a reduction of the asymmetry with respect to visual attention between the hemispheres leading to changes of the pseudoneglect.

Further studies, however, have also shown that whether pseudoneglect can reliably be measured is also dependent on the stimulus duration. Studies showing the stimuli only for a small duration (such as 150 ms or 1000 ms) have been able to reliably measure a pseudoneglect with a change in age, while in studies in which no temporal restriction have been given have not always been able to do so (Schmitz and Peigneux 2011; Benwell et al. 2014; Brooks et al. 2016).

By now, landmark task and line bisection were systematically investigated with respect to effects of aging. We add a more naturalistic free visual exploration task that resembles more closely everyday exploration and investigate whether age modulates the asymmetries in free visual exploration.

The goals of the present study were threefold: (a) to assess the temporal dynamics of pseudoneglect during visual exploration of naturalistic everyday scenes; (b) to investigate the influence of age on pseudoneglect; and (c) to determine whether pseudoneglect, as assessed by a classical paper–pencil task, would correlate with pseudoneglect observed in a free visual exploration task. To this end, we tested 60 healthy participants, ranging from young adults to elderly (i.e., 22–86 years of age). Furthermore, we were interested in whether other factors such as gender, handedness, and subjective alertness would modulate visual exploration patterns.

## Methods

### Participants

Sixty neurologically healthy adults participated in this study (age range 22–86 years, 31 women, see Table 1). Participants gave their written informed consent prior to participation. The study was carried out in accordance with the code of ethics of the World Medical Association (Declaration of Helsinki). All participants had normal or corrected-to-normal visual acuity, and participants with a history of eye diseases were excluded from the study. None of the subjects reported any difficulties to clearly perceive the visual stimuli while performing the experimental tasks.

**Table 1:** Overview of demographic data of the 60 participants included in the study

	Mean ± SD	Range
Age (years)	43.05 ± 19.60	22–86
Education (years)	17.19 ± 2.99	8–22
Subjective alertness	7.36 ± 1.84	2–10
Handedness	88.3% (*N* = 53) right-handed	
Gender	51.7% (*N* = 31) females	

### Stimuli and materials

#### Free visual exploration task

**Fig. 1:**
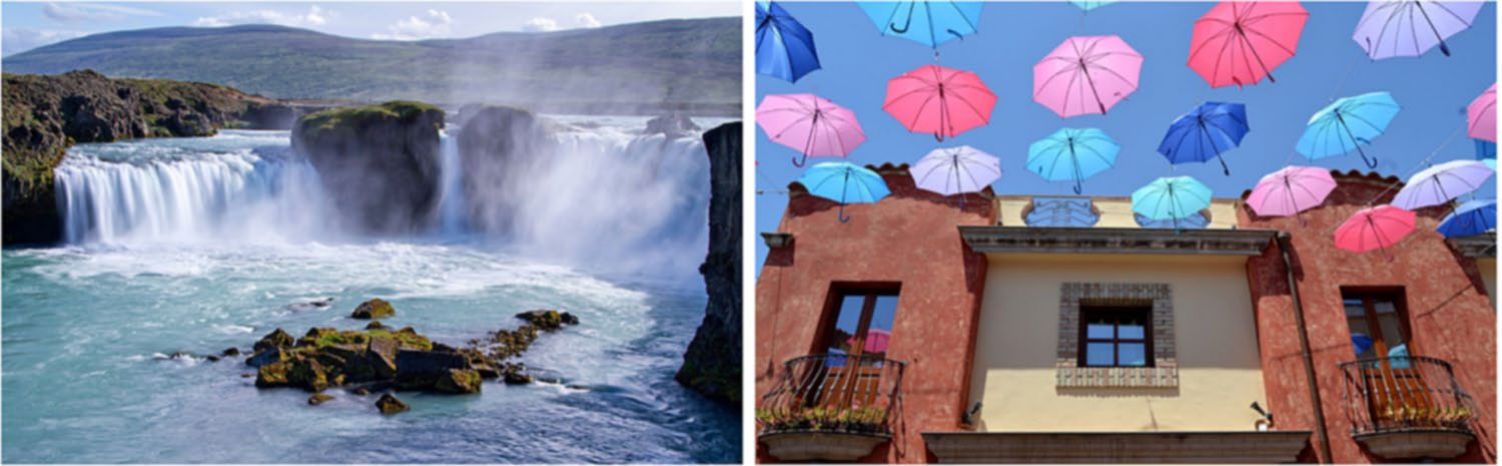
Example of stimuli used in the free visual exploration task

In the free visual exploration task, participants viewed a series of naturalistic, coloured photographs of everyday scenes (*N* = 120) in a dimly lit room, while their gaze was recorded by means of a contact-free eye-tracking system (see section Eye tracking below for further details). The images were selected from a free image database (pixabay.com), from the sub-categories “nature” and “architecture”. The selection of the images was based on their saliency maps, as assessed by a dedicated algorithm (Itti et al. 1998; Paladini et al. 2017). This algorithm takes into account different characteristics of the features within an image, such as orientation, colour, and intensity, which allow the computation of a map of salient regions within the image. This procedure allowed to balance the overall saliency between the left (*M* = 31.6, SD = 7.37) and the right (*M* = 32.3, SD = 8.49) halves of the images (*t*(119) = − 0.945, *p* = 0.347). Please note that the raw values produced by the algorithm were multiplied by 100 to increase the readability of the results. Moreover, images containing humans or letterings were not included. Two examples of presented photographs are shown in Fig. 1 and the exhaustive choice of the experimental stimuli as well as the ratings produced by the saliency algorithm (Itti et al. 1998) are available at the URL: https://osf.io/zd3qm/. To avoid fatigue and to allow for periodical calibration of the eye-tracking system, the photographs were distributed into six sets of 20 photographs each. Following a nine-point calibration, the free visual exploration task proceeded by displaying the series of images, one at a time, in a random order. After each set (20 images), participants were allowed to take a short break and the calibration was repeated. Each trial began with a central fixation marker (1.5 s), followed by an image displayed for 7 s. Participants were instructed to freely explore the images, as if they were looking at photographs in a photo album

#### Line bisection task

In a classical line bisection task, participants were presented with twenty horizontal lines of different lengths, printed on a landscape A4 paper sheet; the actual centre of each line varying along the horizontal dimension (Schenkenberg et al. 1980). The centre of the paper sheet was aligned with the participant’s midsagittal plane, and participants were instructed to bisect all lines as quickly as possible using their dominant hand. The uppermost and the lowermost lines were used as practice trials. For the remaining 18 lines, the deviation of the bisection mark from the actual centre of the line was measured in cm. This value was further divided by the actual midline (in cm) of the respective lines, and then multiplied by 100, thus yielding a percent deviation; thereby, negative values indicated a left-sided deviation, and positive values a right-sided deviation.

#### Subjective alertness assessment

The subjective level of alertness was assessed by means of a visual analogue scale (VAS). On a 10 cm long vertical line, ranging from “very alert” to “not at all alert”, participants were instructed to draw a horizontal mark to indicate how alert they felt. The distance between the lower extreme of the vertical line and the participants’ mark was measured in mm, with lower values indicating a subjective lower level of alertness.

#### Handedness

Handedness was assessed by the Edinburgh Handedness Inventory (EHI; Oldfield 1971), measuring hand preference by asking participants to choose which hand(s) is used for a range of 10 everyday tasks. The EHI scores range from − 100 to 100, with negative scores indicating a tendency to left-handedness, and a positive score indicating a tendency to right-handedness.

### Eye tracking

In the visual exploration task, participants viewed a series of images that were presented full-screen on a 22″ computer display (Dell, Dell Inc.), with a refresh rate of 60 Hz, a colour-depth of 32 bit, a resolution of 1680 × 1050 pixels, and subtending a visual angle of approximately 37.48° × 23.80°. The screen was placed at the eye level, in line with the participants’ midsagittal plane and participants were seated approx. 65 cm from the screen. A contact-free eye-tracking system, equipped with automatic head-movement compensation, was used to record eye movement data (RED 250, SensoMotoric Instruments GmbH). The eye position was sampled at 250 Hz, with a spatial resolution of 0.03° and an average gaze accuracy of 0.4°. Stimulus presentation was controlled by the Experiment Center software (SensoMotoric Instruments GmbH), and the iViewX software (SensoMotoric Instruments GmbH) was used for eye movement data acquisition. Raw data were parsed into fixations and saccades using the default parameters of the manufacturer’s analysis software (BeGaze™, SensoMotoric Instruments GmbH). The results were exported in an open format (.txt) and were analyzed using R (Version 3.5.0) and Matlab 2019b (The MathWorks Inc., Natick 2019).

### Data analysis

To ensure that all scan paths would indeed start from the middle of the images, as enforced by the central fixation cross presented before each image, an offline drift correction was performed. For this purpose, a pixel band of 184 pixels, corresponding to 2° visual angle, around the vertical midline of the image was defined. Images in which the initial fixations started outside of this pixel band were excluded from further analysis (i.e., 472 out of 7159 images). For the remaining fixations, an offline drift correction was applied. To this end, the horizontal deviation from the midline on the *x* axis was calculated for the last fixation taking place on the fixation cross. Afterwards, all the *x* values of the fixations of the following trial were shifted by this offset. At last the mean number of fixations as well as the mean fixation duration, were calculated.

To analyse the time course of attentional asymmetries (Nuthmann and Matthias 2014), the average gaze position deviation was computed over 10 ms bins, i.e., *N* = 700 for 7 s (Hartmann et al. 2019). In brief, the horizontal deviation from the centre of the image, i.e., the difference between × coordinates of the corresponding fixations and the midline, was calculated for every fixation falling within a given 10 ms bin; the values were then averaged within the corresponding bin. This served as a measurement of attentional asymmetries (Hartmann et al. 2019). Thus, negative values indicate a leftward bias, and positive values a rightward bias. Deviation values were computed for each participant and every time bin. The nonparametric random permutation procedure proposed by Maris and Oostenveld (2007) was implemented to account for the problem of multiple comparisons. With this approach, time bins during which the gaze position could be predicted by the age or the performance in the line bisection task, were defined and tested for significance. Specifically, it was tested for each 10 ms bin whether age or the performance in line bisection was a significant predictor for the horizontal gaze position. Adjacent 10 ms bins for which a significant predictor (*p* < 0.05) was found formed a cluster, and Fisher’s *F* values of all bins within a cluster were summed up, resulting in “cluster mass values”. These values were then compared to a “random distribution” of mass values that was obtained by computing the highest “by chance significant” cluster mass value from randomly permutated bins for 5000 times. The *p* value of each initial cluster was then obtained from the position of the cluster mass value within this “random distribution” (see Hartmann et al. 2019). In a next step, the horizontal gaze position was averaged for the time period of significant clusters and correlated with other variables of interest (Spearman’s correlations are reported). The permutations, as well as the corresponding *p* values, were obtained using the R-package “permuco” (Frossard and Renaud 2018).

Moreover, to test whether additional factors such as gender or subjective alertness would influence the visual exploration behaviour, a linear mixed model with factors age, performance in the line bisection, handedness, gender and subjective alertness was calculated. For this analysis, the average gaze position over the whole presentation time was considered, irrespective of its time-course.

## Results

### Free visual exploration pattern

**Fig. 2:**
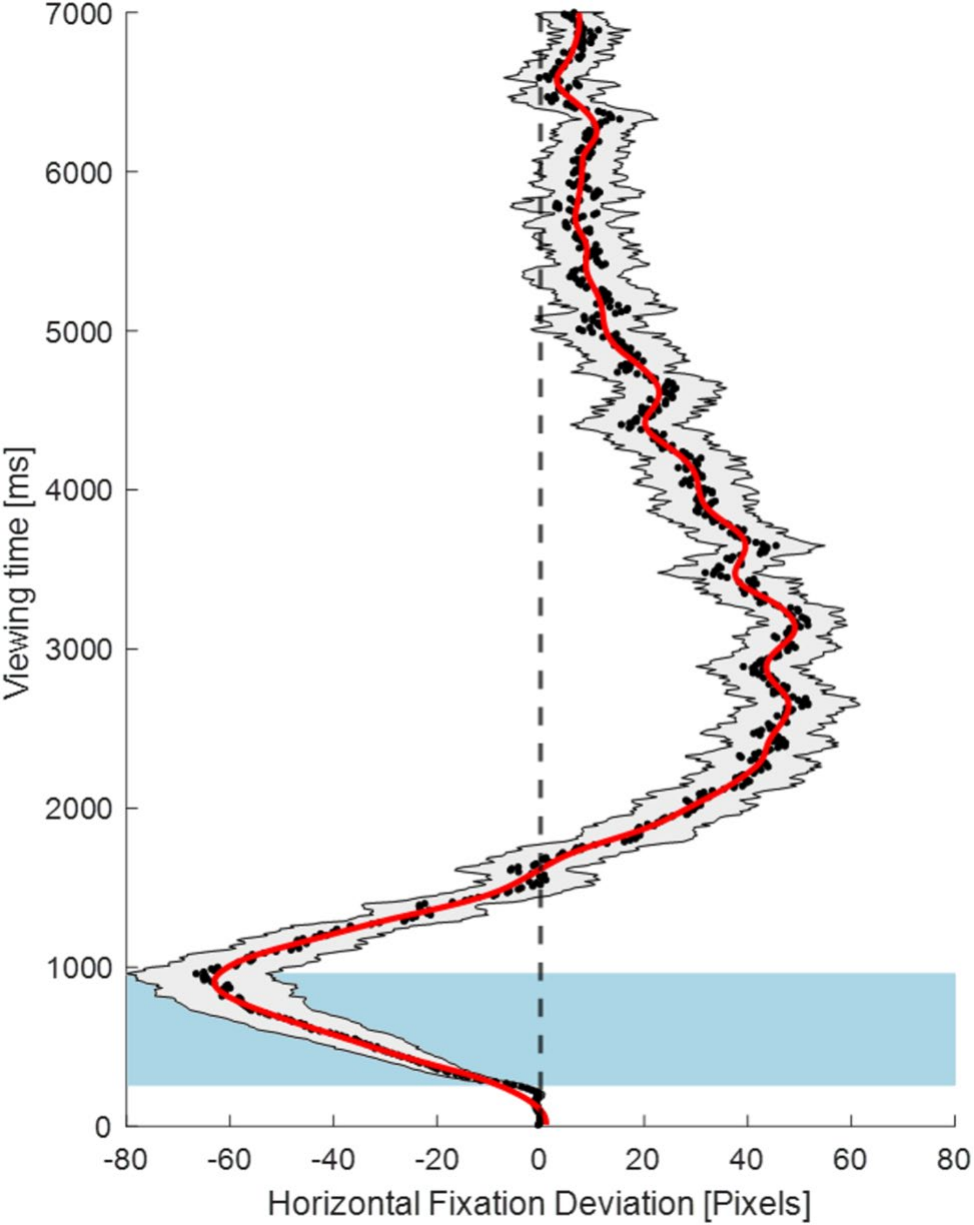
Time-course of the exploration pattern, averaged across all images and all participants. The grey area around the smoothed red line represents the standard error of the mean of the averaged gaze position. The blue box represents the time-window during which age was a significant predictor for the average gaze position

Participants produced on average 21.41 fixations per image (*M* = 21.41, SD = 4.37), with an average gaze fixation duration of 250 ms (*M* = 250.41 SD = 43.65). Overall, during the initial stages of the exploration, there was a tendency to deviate towards the left side of the image. This initial leftward bias lasted for about 1.5 s, after which the exploration pattern shifted towards the right side of the image. It is worth to note that the maximal deviation from the midline was more pronounced for the left than for the right part of the images, even though overall, participants spent more time on the right than on the left side of the images (Fig. 2).

### The effect of age on spatio-temporal asymmetries in the free visual exploration task

**Fig. 3:**
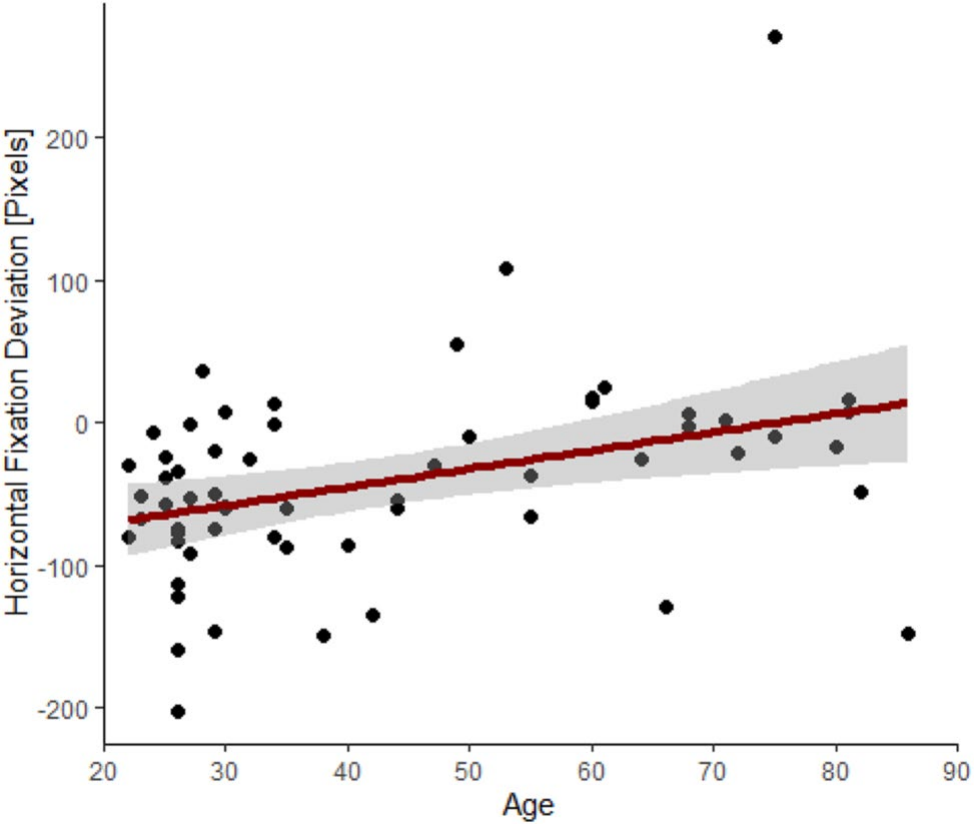
Correlation between age and the average horizontal gaze deviation in pixels between 260 and 960 ms, indicating attenuation of the leftward bias with increasing age

The nonparametric random permutations indicated a significant time cluster at 260–960 ms (cluster mass = 632.6, *p* = 0.0272, see Fig. 2). During this early phase of visual exploration, age modulated the exploration behaviour in a way that, with increasing age, the initial leftward bias was attenuated (*r*_*S*_(60) = 0.38*, p* = 0.003, see also Fig. 3).

### Visuo-spatial asymmetries in the line bisection task

We further investigated whether spatial asymmetries in a more naturalistic free visual exploration task would correlate with the performance in a classical test of visuo-spatial attentional bias, namely, the line bisection task.

**Fig. 4:**
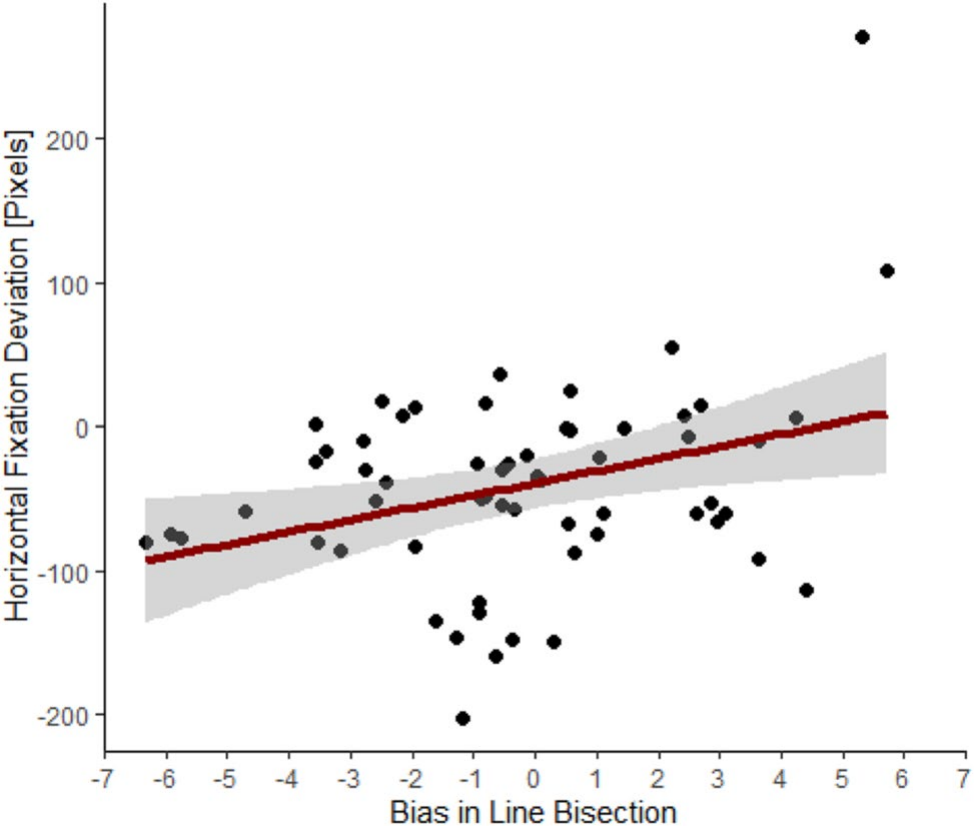
Correlation between the bias in the line bisection task (percentage deviation from the actual midline) and the average horizontal fixation deviation in the free visual exploration task during the time interval between 300 and 1490 ms; this significant correlation indicates that a stronger leftward bias in the line bisection task correlated with a stronger leftward deviation in the free visual exploration

Overall, the performance in the line bisection task indicated a small leftward bias (relative deviation from the middle: *M* = − 0.27%; 95% CI [− 0.97, 0.43]; *SD* = 2.69%, range − 6.33–5.71%). The nonparametric random permutations indicated a significant time interval between 300 and 1490 ms (cluster mass = 795.87, *p* = 0.0218), in which the performance in the line bisection task was positively correlated with the average gaze position (*r*_*S*_(60) = 0.27*, p* = 0.034, see Fig. 4). As such, performance in the line bisection task was predictive of the mean gaze position in the visual exploration task during the initial phase of exploration.

Furthermore, there was no significant correlation between age and the performance in the line bisection task (*r*(60) = 0.19, *p* = 0.136).

### Influence of other individual factors on the performance in the free visual exploration task

A linear mixed model was calculated to test whether, in addition to age and performance in the line bisection task, other factors such as gender, handedness, and subjective alertness would influence the exploration behaviour, as measured by the average gaze position. In line with previous analyses, line bisection performance (*p* = 0.028) and age (*p* = 0.035) modulated the average gaze position in the visual exploration task. Yet, none of the additional factors had a significant influence (*p* = 0.92 for gender, *p* = 0.38 for handedness, and *p* = 0.62 for subjective alertness).

## Discussion

The aim of the present study was to investigate the spatial and temporal dynamics of free visual exploration of complex naturalistic images. Furthermore, we were interested in assessing whether different individual factors such as age, gender, handedness, and subjective alertness would modulate these spatio-temporal dynamics. In agreement with previous studies (Nuthmann and Matthias 2014; Hartmann et al. 2019), we found a pseudoneglect, as manifested by a leftward bias during the initial phase of the free visual exploration of an image. Second, and more importantly, we found a significant influence of age during a critical time window in the early phase of exploration (between 260 and 960 ms), i.e., this leftward bias was attenuated with increasing age. Thirdly, we found a significant correlation between the line bisection bias and the spatial bias in free visual exploration, indicating that a stronger leftward bias in the line bisection task correlated with a stronger leftward deviation in the visual exploration task. Finally, we found no significant effect of subjective alertness, handedness, or gender on spatio-temporal fixation dynamics during free visual exploration.

To the best of our knowledge, a critical time window during which age-dependent differences in spatial biases are evident in free visual exploration has never been described before in the literature. A leftward bias in the visual exploration behavior is reminiscent of the phenomenon of pseudoneglect. The so-called pseudoneglect is a slight leftward attentional bias, commonly observed, e.g., in the line bisection task. Several studies yielded evidence for an age-related reduction of pseudoneglect, i.e., from a strong attentional leftward bias in young adults to a suppressed or even reversed bias in the elderly, as in our study (see Schmitz and Peigneux 2011 for a review). However, the literature is not conclusive, i.e., several other studies failed to show such age-related changes in spatial biases (Beste et al. 2010; Hatin et al. 2012; Brooks et al. 2016). The results of our study suggest that time is a critical factor, i.e., an age-dependent modulation of spatial biases is only evident in a critical time window; this might explain, at least in part, the discrepancies in the earlier literature.

The null results between age and the line bisection underlie the fact that assessing leftward biases within specific time windows, in line with a characterization of dynamic behaviour with a high temporal resolution, is necessary.

Only few studies examined the influence of age on visual exploration behaviour. Urwyler and colleagues (Urwyler^ 2015^) analysed the influence of age on visual exploration during driving. They found an effect of age, showing that older participants had a narrowed visual exploration field. Furthermore, detection of targets in a visual search task decreased with age, especially for more peripheral targets (Gruber^ 2014^). However, to the best of our knowledge, our study is the first to use a free visual exploration paradigm in participants of different ages.

The origin of the age-related modulation of spatial biases is still debated. It has been suggested that healthy aging might be associated with a functional decline of the right hemisphere, coupled with a left-hemispheric compensation (Dolcos et al. 2002; Schmitz and Peigneux 2011). Indeed, an age-related reduction of the right-hemispheric lateralization has been shown in an EEG study applying a landmark task (Learmonth^ 2017^). Such a relative hyperactivity of the left hemisphere would explain the rightward bias shift in older individuals. This phenomenon can be considered as a less pronounced form of the biased spatial dynamics that have been described in classical neglect models (e.g., Kortman and Nicholls 2016; Delazer^ 2018^). An alternative explanation may be a decline in corpus callosum function, which could impair interhemispheric connectivity. This could then reduce the inhibitory influence that the right hemisphere exhibits in elderly, which would then result in a stronger involvement of the left hemisphere (Schmitz and Peigneux 2011). It has also been proposed that, in elderly individuals yielding comparable behavioural performances as younger adults, the age-related neuronal decline is counteracted by means of plastic reorganization mechanisms (Cabeza^ 2002^). These plastic reorganization mechanisms seem not to take place in all (or at least not to the same extent) elderly individuals. This could, in turn, explain the age-related increase in variability of the free visual exploration pattern our study.

A possible explanation for the critical time window identified by our study, in which age-dependent differences in the visual exploration task were evident, stems from electrophysiological studies. Störmer et al. (Störmer 2013) investigated neural correlates of age-related differences in spatial attention using event-related potentials (ERPs). They found that healthy aging affects attentional selection (supporting the resolution of competition between visual information) at early stages of attentional modulation. To this end, they showed that older adults showed less pronounced selective attentional modulation in the early phase of the visual P1 component (100**–**125 ms) than younger adults. However, with a 25 ms delay relative to younger adults, older adults showed distinct processing of targets (125–150 ms), i.e., a delayed yet intact attentional modulation. Moreover, the magnitude of the delayed attentional modulation was related to the behavioural performance in older adults. Further ERP studies on attention in young (Foxe et al. 2003; Longo 2015) and older adults (Learmonth et al. 2017) also indicated a critical time-window, starting as early as 139 ms after the stimulus presentation. This effect was observed until 400 ms after the stimulus onset. In addition, an age-dependent time window, ranging from 280 to 400 ms, has been reported (Learmonth et al. 2017), which also coincides with the start of the critical time-window in the present study.

In our study, other factors such as subjective alertness, handedness, or gender did not significantly influence the observed spatial bias. Contrary to the previously mentioned studies, subjects in our study were not specifically recruited to increase variability in the aforementioned factors, which led to a balanced cohort, with only a limited variance with regards to subjective alertness as well as handedness.

In conclusion, our study revealed that, during visual exploration of naturalistic everyday scenes, there is a critical time window within the first second of visual exploration in which age is a predictor of the attenuation of this leftward bias. Furthermore, a significant correlation between line bisection bias and spatial bias during visual exploration was found, i.e., the stronger the leftward bias in line bisection, the stronger the leftward deviation during visual exploration. Hence, our study concurs with previous research by providing evidence that free visual exploration of naturalistic scenes generally starts within the left side of an image, but it extends it in two important ways. First, by providing a systematic and detailed time-course investigation of spatial asymmetries during naturalistic scene perception; second, by directly comparing the outcome of two attentional tasks of visuo-spatial nature (i.e., free visual exploration and line bisection task) in a sample of neurologically healthy subjects ranging from young adults to elderly.

## Data Availability

The data used for the analyses*,* as well as experimental stimuli used in the visual exploration task and their saliency maps computed by a dedicated algorithm (Itti et al. 1998) are available at the URL: https://osf.io/zd3qm/.

## Code availability

The code will be made available upon request.
